# Supplementary material for: A novel generic dictionary-based denoising method for improving noisy and densely packed nuclei segmentation in 3D time-lapse fluorescence microscopy images
Source: Sci Rep. 2019 Apr 4;9:5654. doi: 10.1038/s41598-019-41683-3 (PMC6449358; doi:10.1038/s41598-019-41683-3)
Supplement: Supplementary file 1 — Supplementary information [file 41598_2019_41683_MOESM1_ESM.pdf]

Supplementary Information

A novel generic dictionary-based denoising method for improving noisy and densely packed nuclei segmentation in 3D time-lapse fluorescence microscopy images

Lamees Nasser[1,2*] **and Thomas Boudier**[3,4]

[1]Sorbonne Université, UPMC Univ Paris 06, UJF, CNRS, IMT, NUS, Image and Pervasive Access Lab (IPAL), 138632, Singapore.
[2]BioInformatics Institute (BII), Agency for Science, Technology and Research (A*STAR), Singapore 138671, Singapore
[3]Walter and Eliza Hall Institute of Medical Research, Parkville, Victoria, Australia.
[4]Department of Medical Biology, University of Melbourne, Parkville, Victoria, Australia.
[*]lameesm@bii.a-star.edu.sg

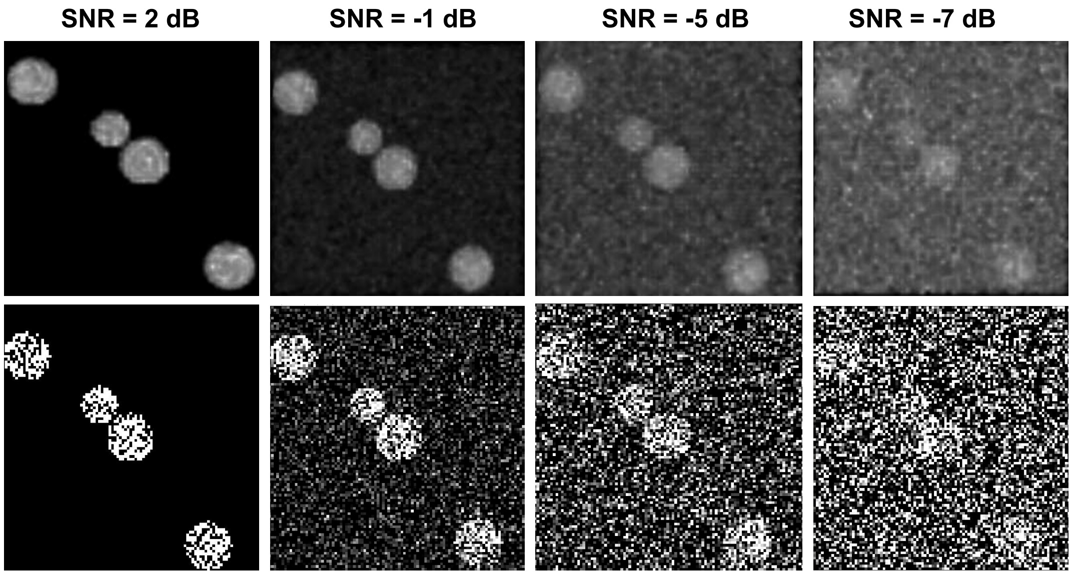

Supplementary Figure 1. Synthetic images with different levels of signal to noise ratio (SNR). Top row: 3D view of the synthetic dataset. Bottom row: single plane (Z = 10) from the synthetic dataset.

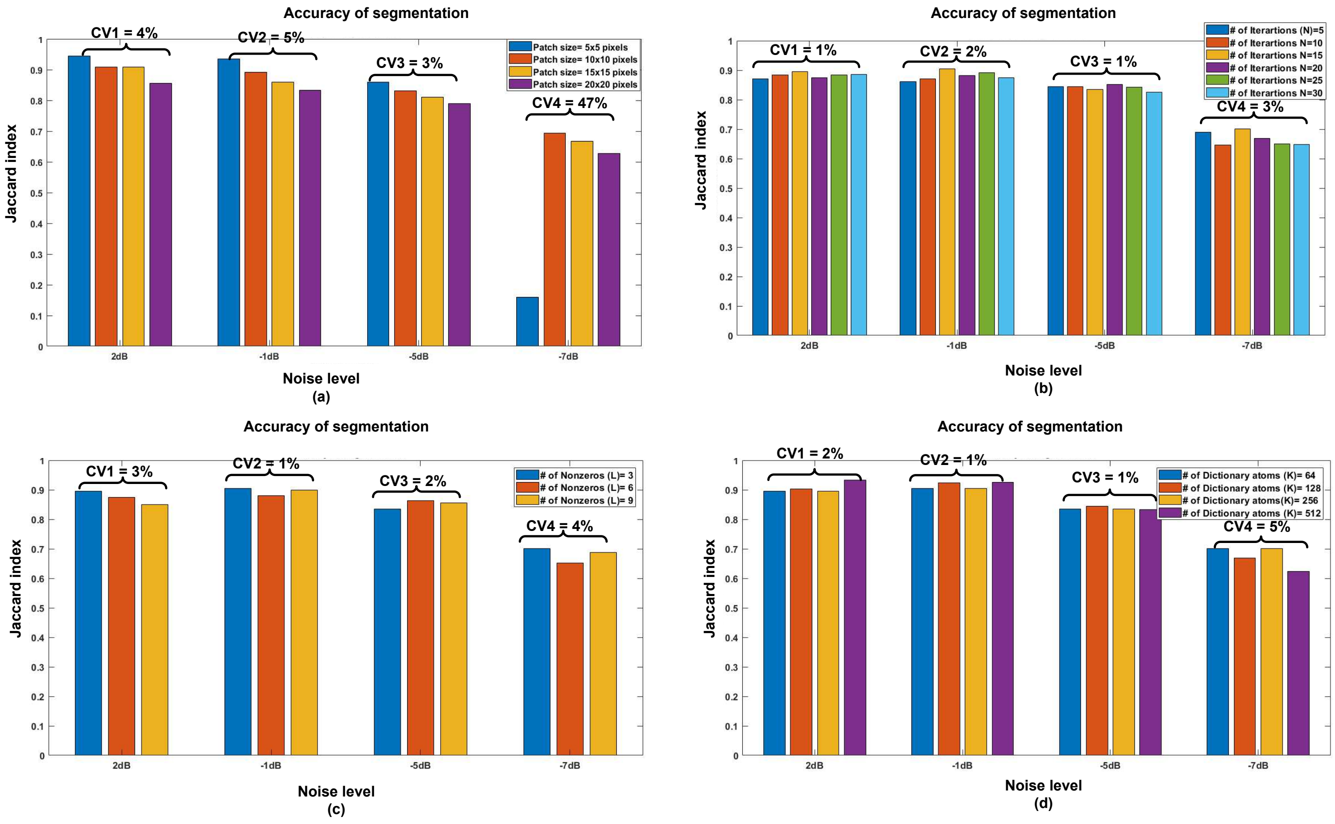

Supplementary Figure 2. Evaluation of the segmentation accuracy with different initialization parameters at different noise levels

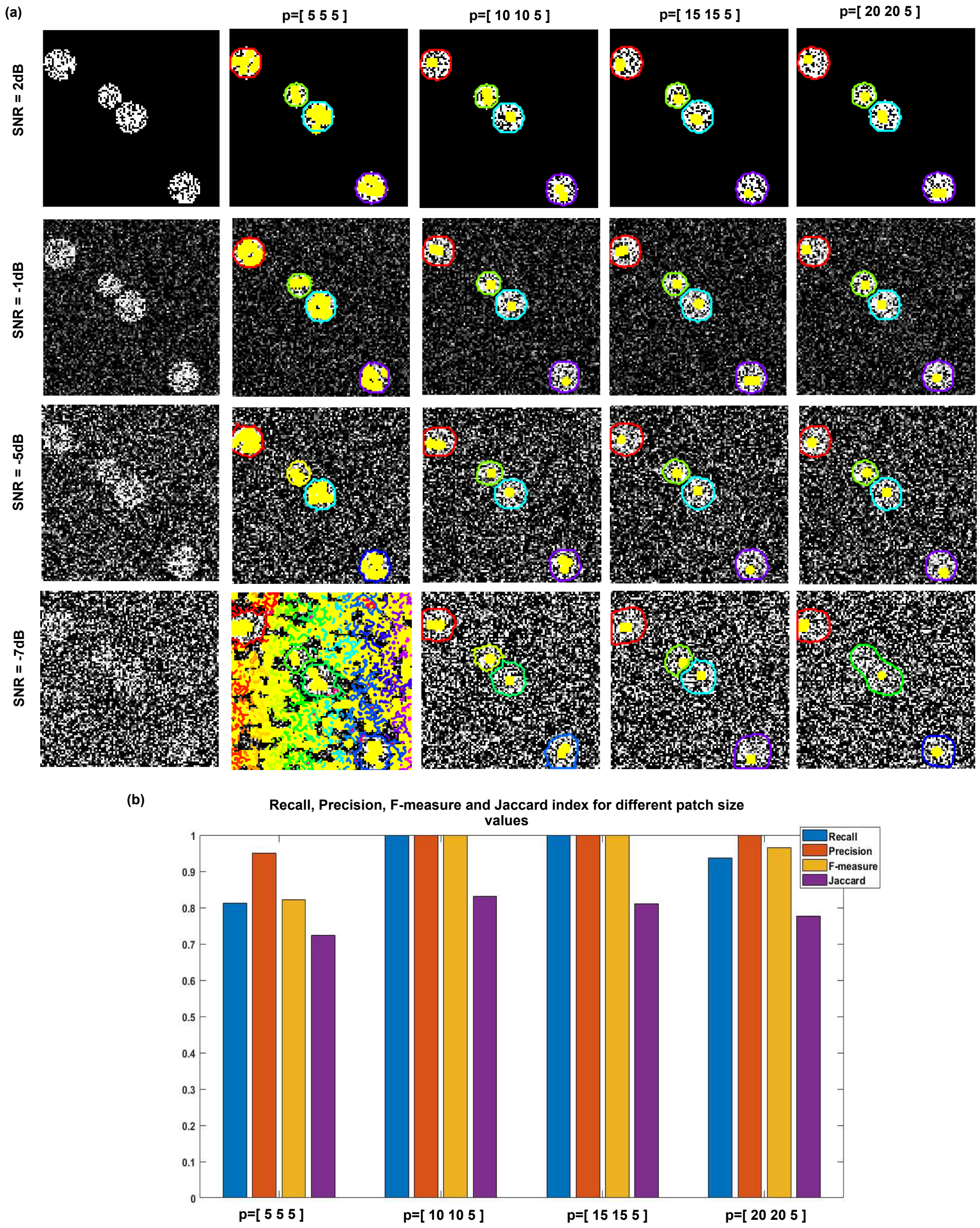

Supplementary Figure 3. Evaluation of patch size values for detection and segmentation results at different noise levels. (a) The results of detection (depicted by yellow dots) and segmentation (delineated by colored contours) overlaid on single plane ($Z = 10$) from synthetic images for different patch size values $p = 5 \times 5 \times 5$, $10 \times 10 \times 5$, $15 \times 15 \times 5$ and $20 \times 20 \times 5$ at various noise levels (SNR $= 2$, -1, -5 and -7 dB). (b) Average Recall, Precision, F-measure, and Jaccard index values of detection and segmentation results at different noise level as a function of patch size.

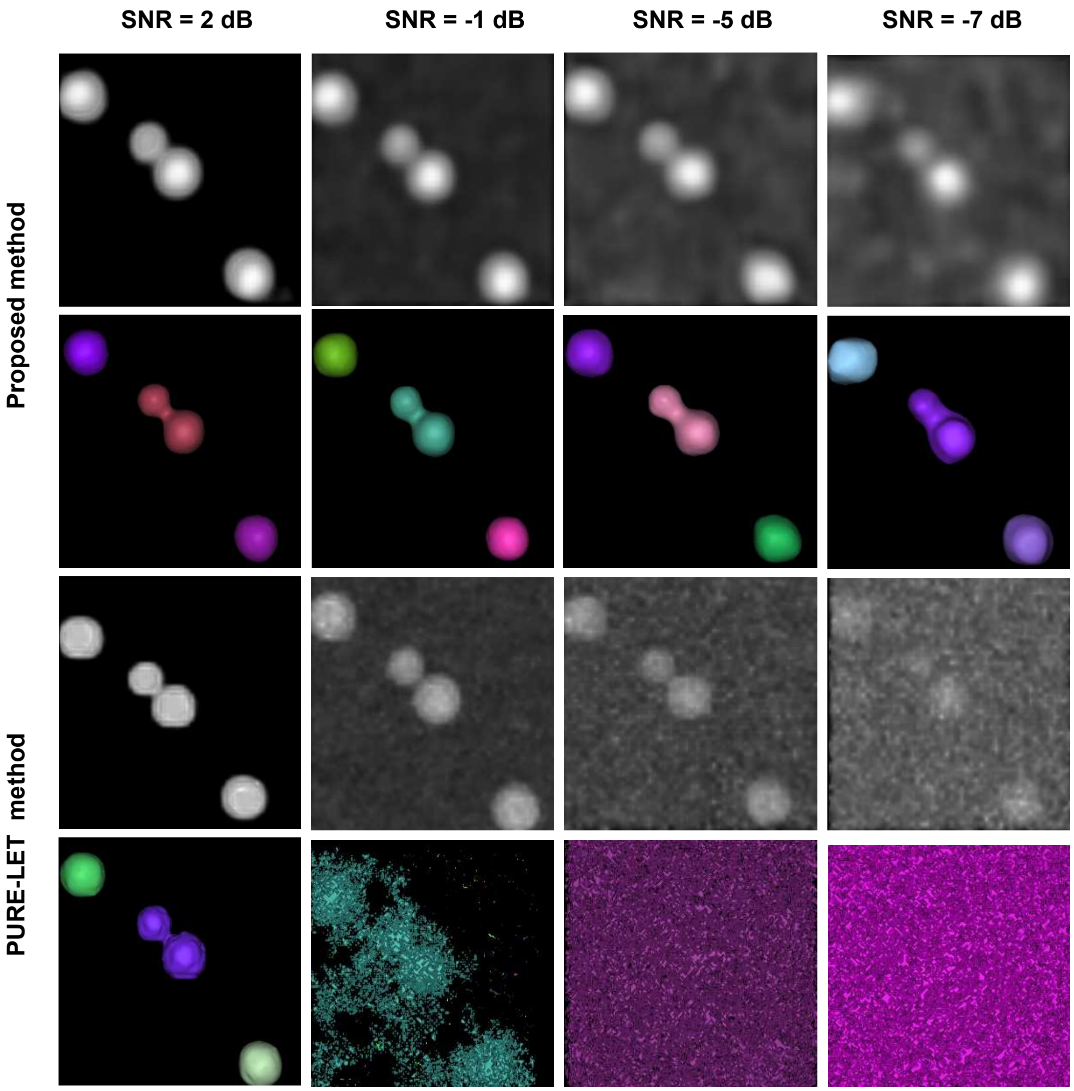

Supplementary Figure 4. A comparison of denoising results on the synthetic dataset (Supplementary Fig 1) using our method and PURE-LET method[1] at different noise levels. First row: 3D view of the denoised images from the proposed method. Second row: 3D view of the segmentation mask of the denoised images from the proposed method. Third row: 3D view of the denoised images from the PURE-LET method. Fourth row: 3D view of the segmentation mask of the denoised images from the PURE-LET method .

Table 1. Segmentation performance of our method (SRS) among various datasets considering the patch size percentage.

	CE-UPMC	Fluo-N3DH-CE (seq1)	Fluo-N3DH-CE (seq2)	Fluo-N3DL-DRO
**Patch**	[20 20 5]	[25 25 5]	[25 25 5]	[10 10 5]
**Approximate average cell nuclei (Pixels)**	7000	10,000	12,000	1,300
**Percentage of patch size (%)**	28	31	26	38
**Recall (%)**	97.9757	96.03	97.49	99.37
**Precision (%)**	98.7743	98.9	98.98	3.32
**F-measure (%)**	98.3476	97.44	98.23	6.43
**Jaccard index (%)**	-	66	70	66.5

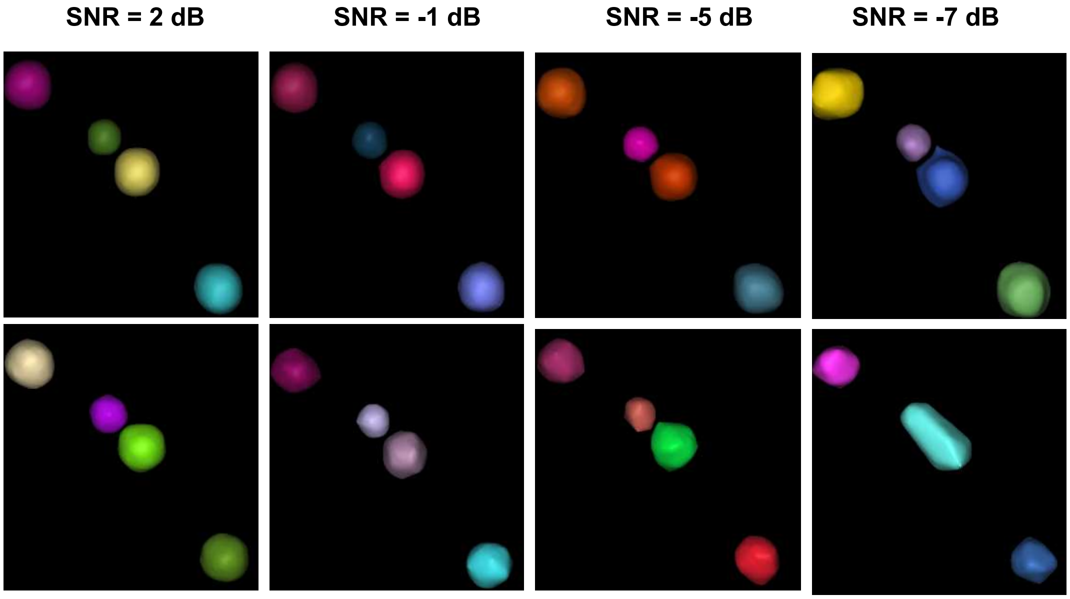

Supplementary Figure 5. A comparison of segemntation results on the synthetic dataset (Supplementary Fig 1) using our method and KTH method[2,3] at different noise levels. First row: 3D view of the segmented images from the proposed method. Second row: 3D view of the segmented images from the KTH method.

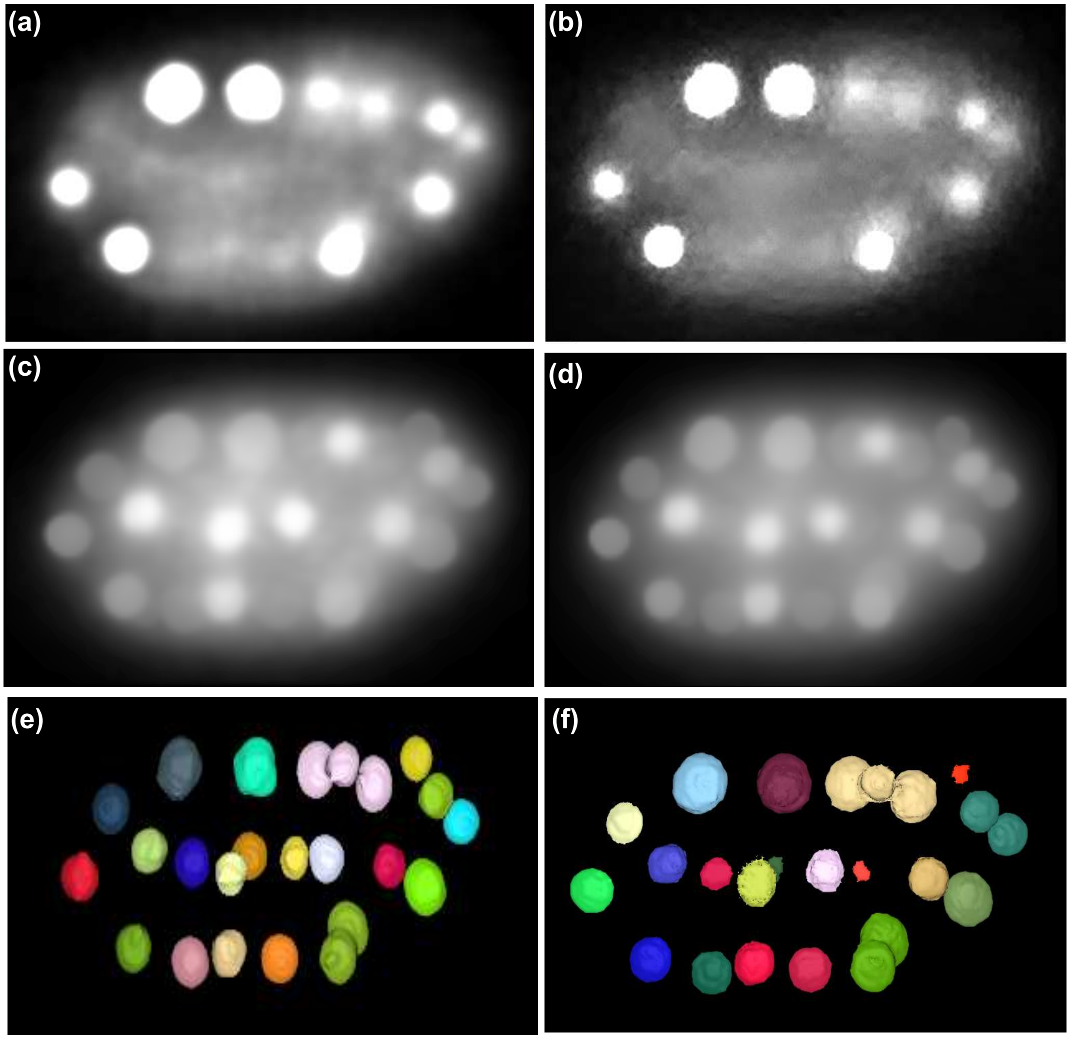

Supplementary Figure 6. A comparison of denoising results on the *CE-UPMC* dataset using our method and PURE-LET method[1] for the same time points as Supplementary Fig 9 (a). First column: shows the results of the proposed method. Second column: shows the results of the PURE-LET method. (a, b) a single plane (Z = 15) of time point (T = 60) for the denoised images. (c, d) 3D view of the denoised images. (e, f) 3D view of the segmentation mask of the denoised images, colours shown are for illustration purpose only, they are not the final segmentation results.

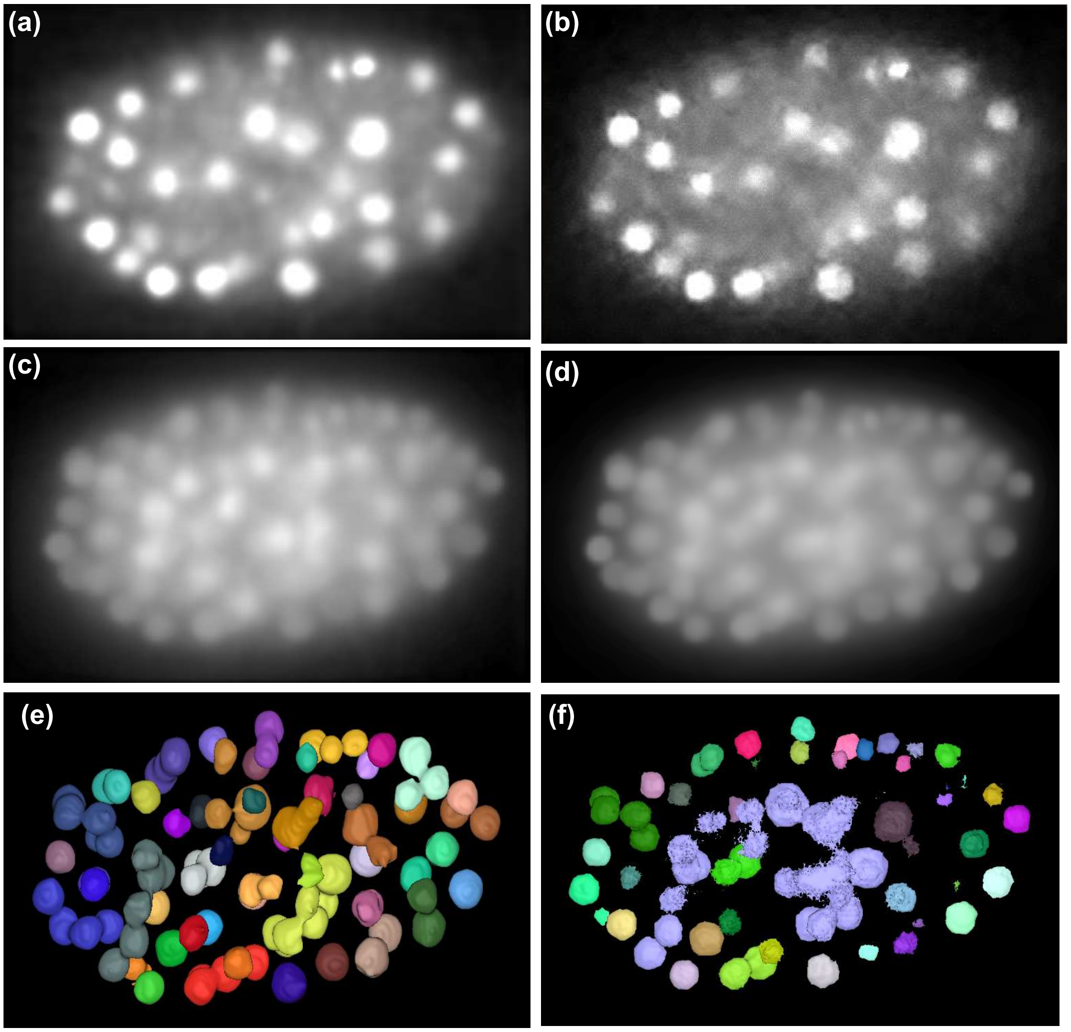

Supplementary Figure 7. A visual comparison of denoising results on the *CE-UPMC* dataset using our method and PURE-LET method[1] for the same time points as Supplementary Fig 9 (b). First column: shows the results of the proposed method. Second column: shows the results of the PURE-LET method. (a, b) a single plane (Z = 15) of time point (T = 140) for the denoised images. (c, d) 3D view of the denoised images. (e, f) 3D view of the segmentation mask of the denoised images, colours shown are for illustration purpose only, they are not the final segmentation results.

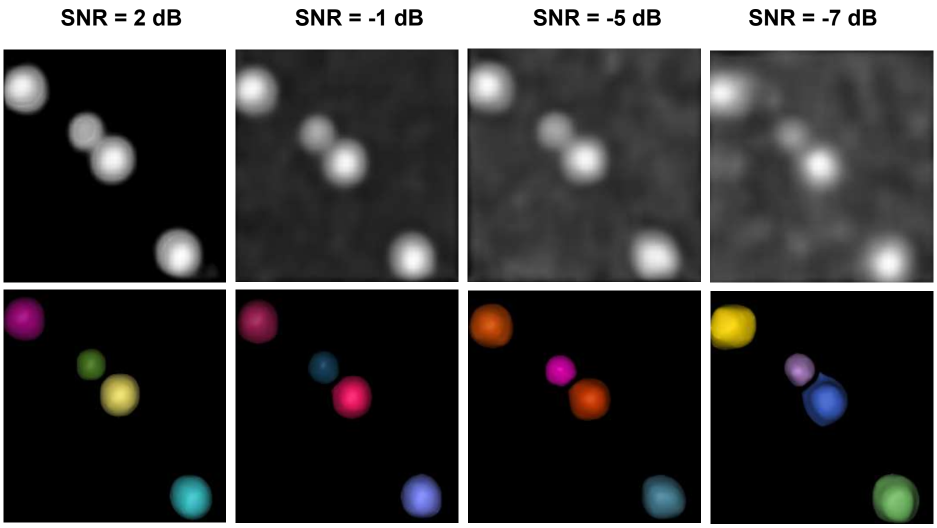

Supplementary Figure 8. Example of denoising and segmentation results on the synthetic dataset (Supplementary Fig 1) at different noise levels. First row: 3D view of denoising result. Second row: 3D view of the segmentation result.

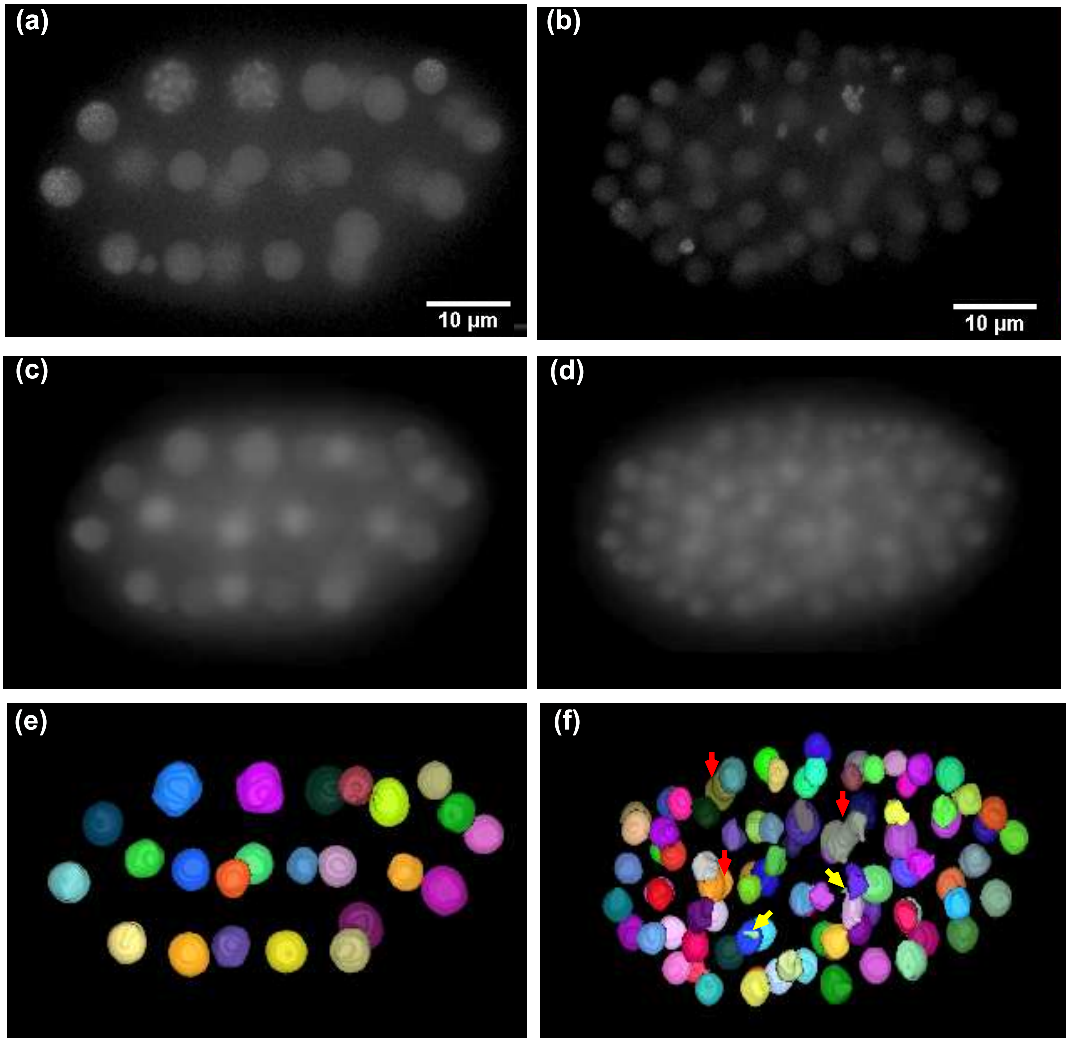

Supplementary Figure 9. Example of denoising and segmentation results on the *CE-UPMC* dataset. (a, b) 3D view of the raw data for time points (T = 60 and 140) respectively. (c) 3D view of the denoising result for (a). (d) 3D view of the denoising result for (b). (e) 3D view of the segmentation result for (c). (f) 3D view of the segmentation result for (d). Note that, yellow arrows indicate noisy objects and red arrows indicate merged cell nuclei.

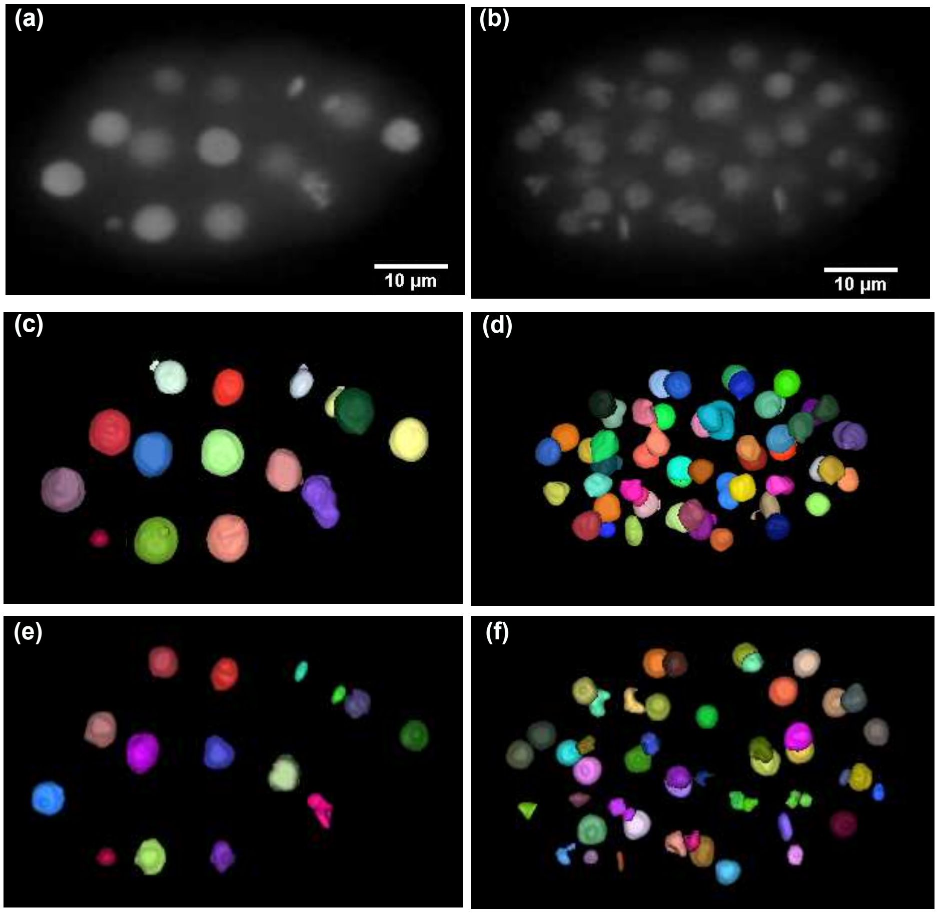

Supplementary Figure 10. An example of the segmentation results for our method and the results from the original paper[4] of *CE-UPMC* dataset at time points T = 40and120 **respectively.** First column: shows results of time point (T = 40). Second column: represents results of time point (T = 120). (a, b) 3D view of the raw data. (c, d) 3D view of our segmentation result. (e, f) 3D view of the segmentation result from the original paper[4] of *CE-UPMC* dataset.

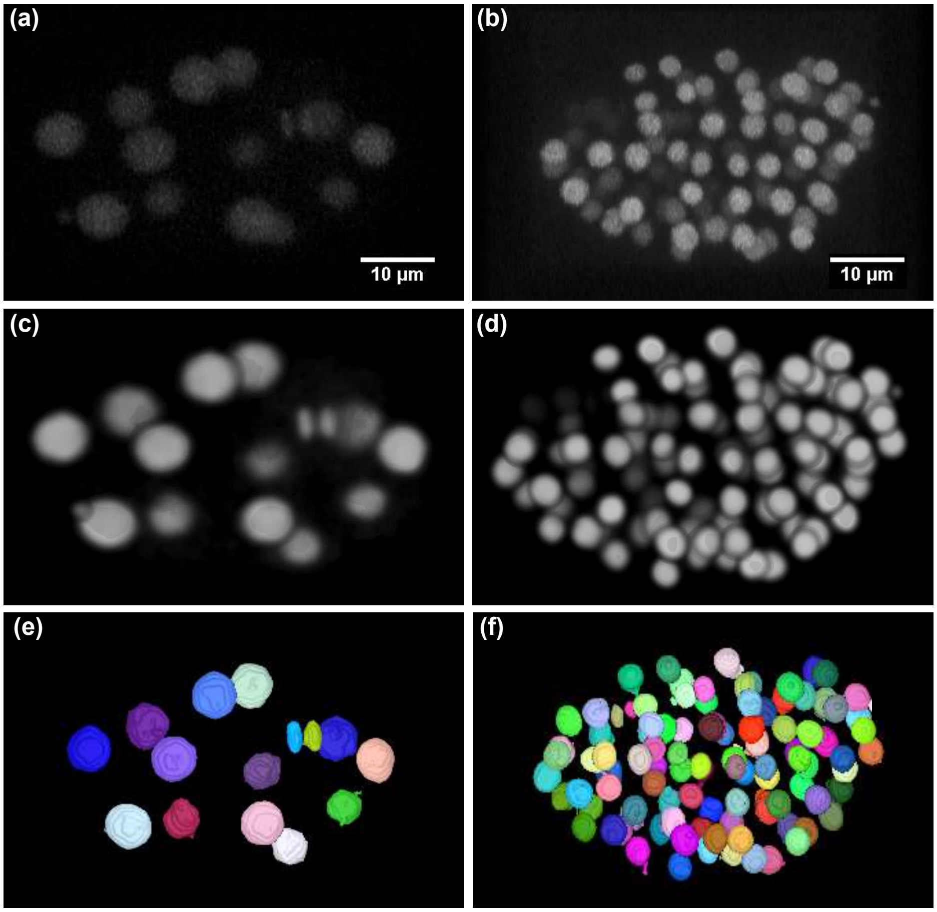

Supplementary Figure 11. Example of denoising and segmentation results on the *Fluo-N3DH-CE* dataset. (a) 3D view of the raw data for time point (T = 28) from sequence (1). (b) 3D view of the raw data for time point (T = 106) from sequence (2). (c) 3D view of denoising result for (a). (d) 3D view of denoising result for (b). (e) 3D view of the segmentation result for (c). (f) 3D view of the segmentation result for (d).

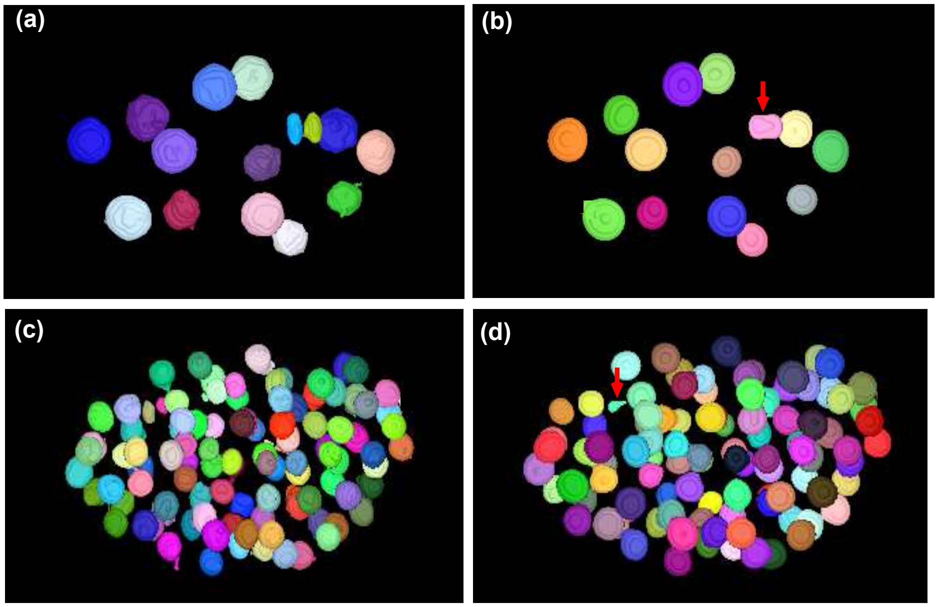

Supplementary Figure 12. A visual comparison of segmentation results over *Fluo-N3DH-CE* dataset using our method and KTH method[2,3] for the same time points as Supplementary Fig 11). First row: shows results of time point (T = 28). Second row: represents results of time point (T = 106). (a, c) 3D view of our segmentation result. (b, d) 3D view of KTH segmentation result.

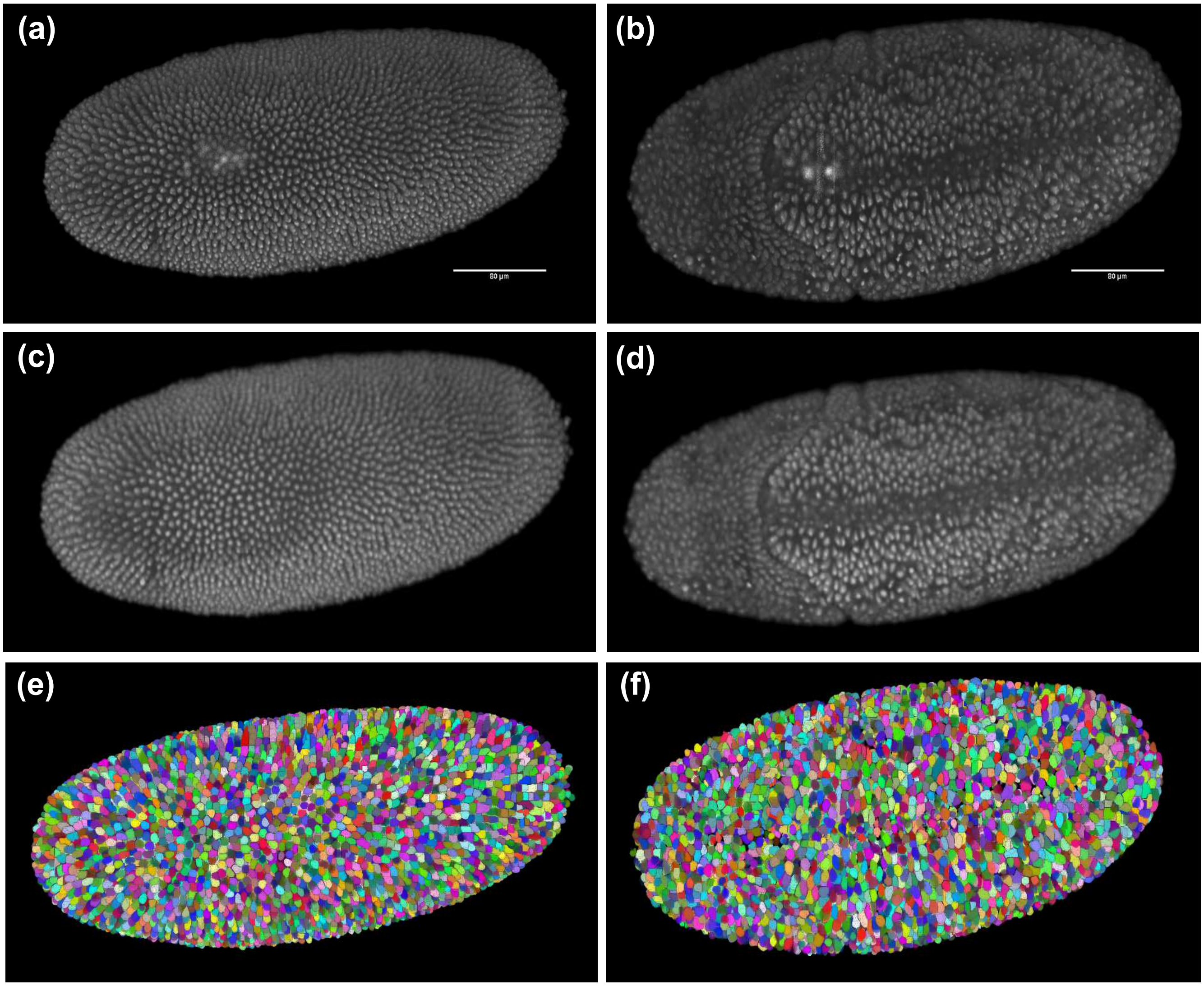

Supplementary Figure 13. Example of denoising and segmentation results on the *Fluo-N3DH-DRO* dataset. (a, b) 3D view of the raw data for time point (T = 0) from sequence1 and sequence2, respectively. (c) 3D view of the denoising result for (a). (d) 3D view of the denoising result for (b). (e) 3D view of the segmentation result for (c). (f) 3D view of the segmentation result for (d).

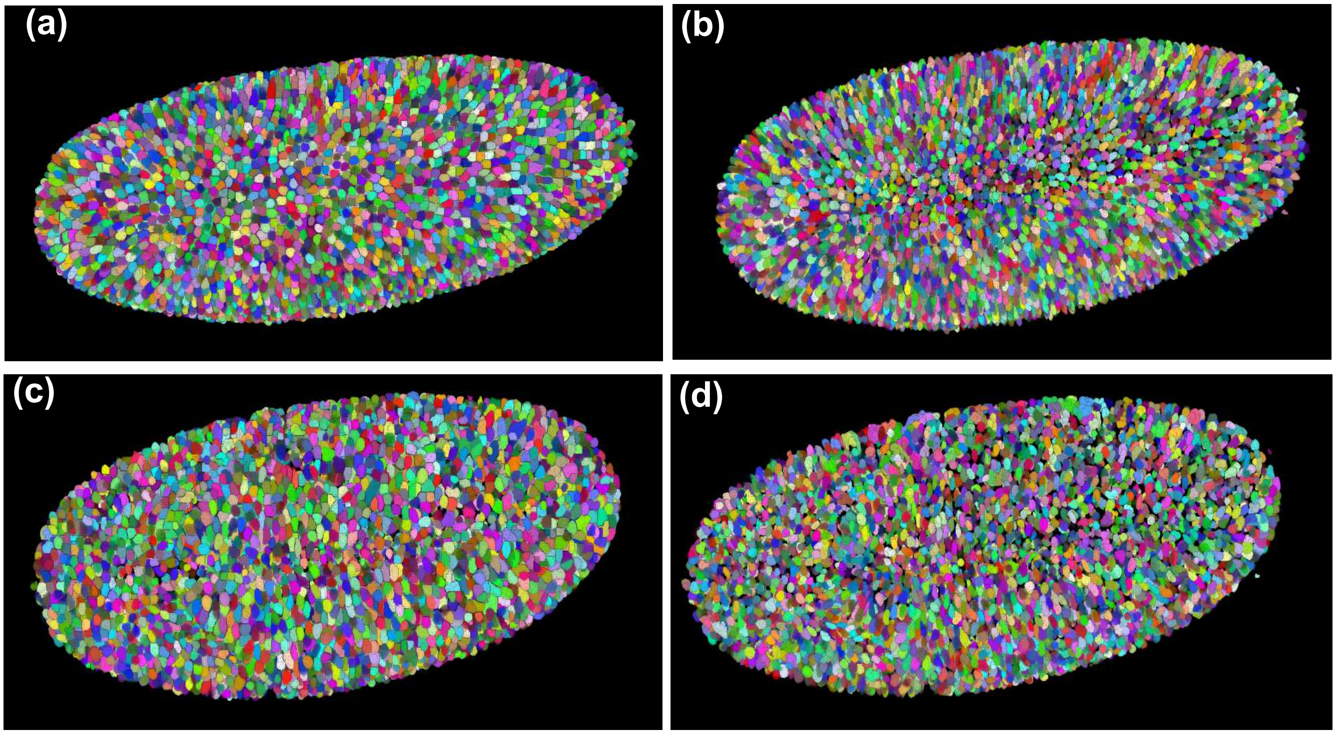

Supplementary Figure 14. A visual comparison of segmentation results over *Fluo-N3DH-DRO* dataset using our method and KTH method[2,3] for the same time points as Supplementary Fig 13). First row: shows results of time point (T = 0) from sequence1. Second row: represents results of time point (T = 0) from sequence2. (a, c) 3D view of our segmentation result. (b, d) 3D view of KTH segmentation result.

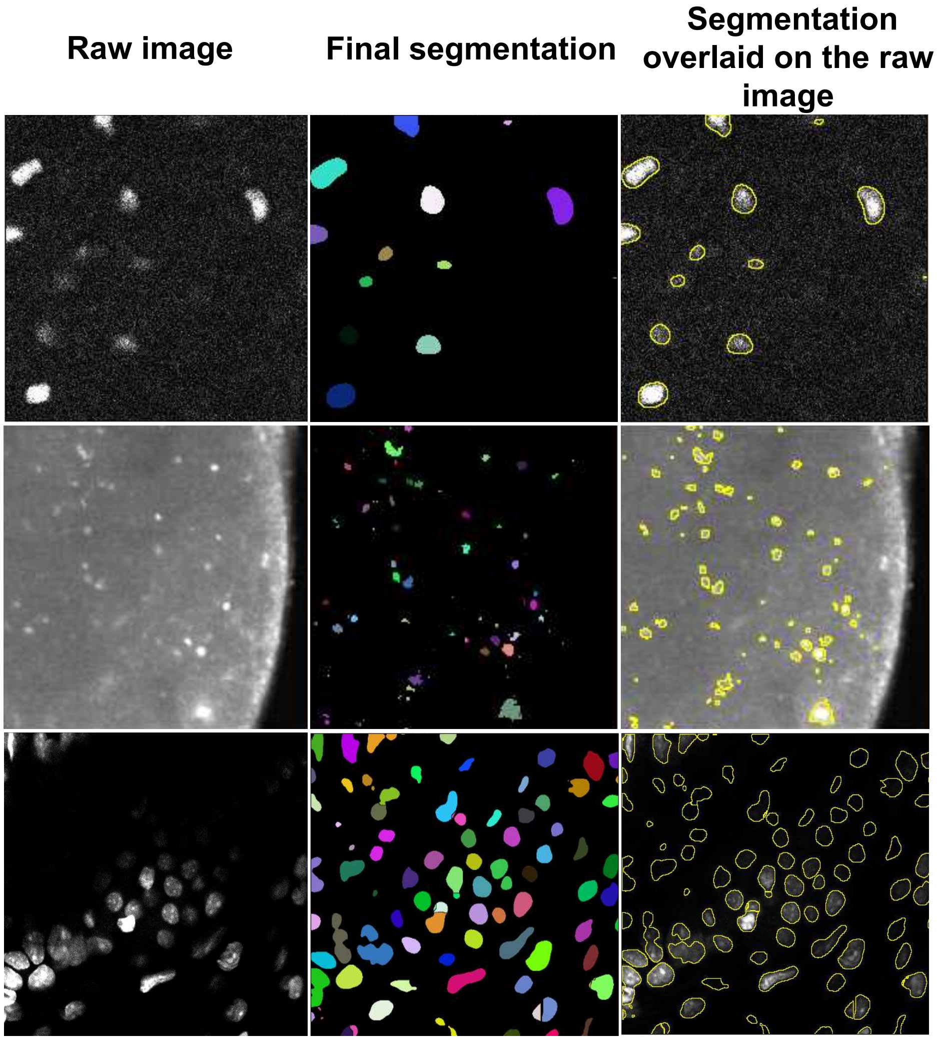

Supplementary Figure 15. A visual example of segmentation results on real datasets coming from various tissues using our method. First raw: thymus tissue (a single plane Z = 106). Second raw: lymphoid tissue (a single plane Z = 204). Third raw: islet of Langerhans tissue[5] (a single plane Z = 100).

References

1. Luisier, F., Vonesch, C., Blu, T. & Unser, M. Fast interscale wavelet denoising of poisson-corrupted images. *Signal Processing* **90**, 415 – 427 (2010).

2. Ulman, V. *et al.* An objective comparison of cell-tracking algorithms. *Nature Methods* (2017).

3. Maška, M. *et al.* A benchmark for comparison of cell tracking algorithms. *Bioinformatics* **30**, 1609–1617 (2014).

4. Gul-Mohammed, J., Arganda-Carreras, I., Andrey, P., Galy, V. & Boudier, T. A generic classification-based method for segmentation of nuclei in 3D images of early embryos. *BMC Bioinformatics* **15**, 9 (2014).

5. Nhu, H. T. T., Drigo, R. A. E., Berggren, P.-O. & Boudier, T. A novel toolbox to investigate tissue spatial organization applied to the study of the islets of langerhans. *Scientific reports* **7**, 44261 (2017).
